# New link between RNH1 and E2F1: regulates the development of lung adenocarcinoma

**DOI:** 10.1186/s12885-024-12392-6

**Published:** 2024-05-24

**Authors:** Wenyue Zhao, Yang Liu, Ying Yang, Liming Wang

**Affiliations:** 1 Department of Thoracic Surgery, The First Hospital of China Medical University, 155# Nanjing North Street, Shenyang, Liaoning China; 2 Department of Pharmacy, The First Hospital of China Medical University, Shenyang, Liaoning China; 3 Department of Operating Room, The First Hospital of China Medical University, Shenyang, Liaoning China

**Keywords:** Lung adenocarcinoma, Ribonuclease/angiogenin inhibitor 1 (RNH1), E2F transcription factor 1 (E2F1), Tumorigenesis

## Abstract

**Background:**

Lung adenocarcinoma (LUAD) is a non-small cell carcinoma. Ribonuclease/angiogenin inhibitor 1 (RNH1) exerts multiple roles in virous cancers. E2F1 is a critical transcription factor involved in the LUAD development. Here, we analyze the expression of RNH1 in LUAD patients, investigate the biological function of RNH1 in LUAD, and demonstrate its potential mechanisms through E2F1 in LUAD.

**Methods:**

In the present study, we presented the expression of RNH1 in LUAD based on the database and confirmed it by western blot detection of RNH1 in human LUAD tissues. Lentiviral infection was constructed to silence or overexpress RNH1 in NCI-H1395 and NCI-H1437 cells. We assess the role of RNH1 on proliferation in LUAD cells by MTT assay, colony formation assays, and cell cycle detection. Hoechst staining and flow cytometry were used to evaluate the effects of RNH1 on apoptosis of LUAD cells. The function of RNH1 in invasion and migration was investigated by Transwell assay. Dual luciferase assay, ChIP detection, and pull-down assay were conducted to explore the association of E2F1 in the maintenance of RNH1 expression and function. The regulation of E2F1 on the functions of RNH1 in LUAD cells was explored. Mouse experiments were performed to confirm the in-vivo role of RNH1 in LUAD. mRNA sequencing indicated that RNH1 overexpression altered the expression profile of LUAD cells.

**Results:**

RNH1 expression in LUAD tissues of patients was presented in this work. Importantly, RNH1 knockdown improved the proliferation, migration and invasion abilities of cells and RNH1 overexpression produced the opposite effects. Dual luciferase assay proved that E2F1 bound to the RNH1 promoter (-1064 ∼ -1054, -1514 ∼ -1504) to reduce the transcriptional activity of RNH1. ChIP assay indicated that E2F1 DNA was enriched at the RNH1 promoter (-1148 ∼ -943, -1628 ∼ -1423). Pull-down assays also showed the association between E2F1 and RNH1 promoter (-1148 ∼ -943). E2F1 overexpression contributed to the malignant behavior of LUAD cells, while RNH1 overexpression reversed it. High-throughput sequencing showed that RNH1 overexpression induced multiple genes expression changes, thereby modulating LUAD-related processes.

**Conclusion:**

Our study demonstrates that binding of E2F1 to the RNH1 promoter may lead to inhibition of RNH1 expression and thus promoting the development of LUAD.

**Supplementary Information:**

The online version contains supplementary material available at 10.1186/s12885-024-12392-6.

## Introduction

Lung cancer is divided into non-small cell lung cancer (NSCLC; ∼85%) and small cell lung cancer (SCLC; ∼15%). NSCLC is further divided into lung adenocarcinoma (LUAD) and lung squamous cell carcinoma (LUSC) [1]. LUAD accounts for approximately 50% of all lung cancer types [2]. LUAD usually presents with significant metastasis, and even patients who may be radically cured have recurrence due to tumor metastasis [3]. Despite improvements in diagnosis and treatment, the 5-year survival rate of LUAD remains less than 20% [4]. Therefore, it is of great significance to find new therapeutic targets for LUAD.

Ribonuclease/angiogenin inhibitor 1 (RNH1) is a member of the protein cytosolic RNase inhibitor family and it can inhibit angiopoietin production [5]. The roles of RNH1 are widely reported in distinct tumors. RNH1 has been showed to inhibit tumor growth and tumor-induced angiogenesis [6]. RNH1 is lowly expressed in colorectal cancer [7] and bladder cancer [8]. X Yao, D Li, DM Xiong, L Li, R Jiang and JX Chen [9] reported that RNH1 suppressed the progression of bladder cancer by regulating epithelial-mesenchymal transition (EMT). RNH1 also hinders the growth of murine melanoma cells and induces apoptosis by reducing angiopoietin [10]. However, RNH1 promotes the resistance of gastric cancer cells to histone deacetylase inhibitors by regulating reactive oxygen species [11]. It has been reported that down-regulation of RNH1 promotes malignant behavior of cancer cells, but the role of RNH1 in LUAD is still unclear. From public databases, we found that the level of RNH1 expression was lower in LUAD tissues than normal tissues (http://gepia2.cancer-pku.cn/). Besides, LUAD patients with high RNH1 expression had better overall survival (OS). Therefore, RNH1 is presumed to affect the development of LUAD.

E2F transcription factor 1 (E2F1), a member of the E2F family, encodes transcription factor in higher eukaryotes, and is involved in cell cycle regulation and DNA synthesis in mammalian cells [12]. E2F1 regulates a variety of cancers at the transcriptional level. Z Lu, RZ Luo, H Peng, M Huang, A Nishmoto, KK Hunt, K Helin, WS Liao and Y Yu [13] reported that E2F1 negatively regulates tumor suppressor genes in breast cancer. Cancer-promoting effect of E2F1 was reported in gallbladder cancer [14] and gastric cancer [15]. Besides, E2F1 transcriptional activity is also required for pancreatic cancer activation [16]. When E2F1-mediated gene repression occurs, cancer cells will undergo apoptosis [17]. Importantly, previous studies reported that E2F1 promotes LUAD cell cycle, activates early LUAD tumorigenesis, and inhibits LUAD cell apoptosis [18–20]. The role of E2F1 in LUAD has been extensively studied, and other factors suppress the progression of LUAD by destabilizing E2F1 or inhibiting E2F1 signaling pathway [21–23]. According to the jasper website (https://jaspar.genereg.net/), E2F1 was predicted to bind to the promoter of RNH1. Additionally, Gene Expression Omnibus (GEO) profiles (www.ncbi.nlm.nih.gov/geoprofiles) showed that E2F1 overexpression caused downregulation of RNH1 expression. Thus, we reasonably speculate that transcriptional factor E2F1 regulates the transcription of RNH1. We focus on RNH1 and aim to investigate the relationship between RNH1 and E2F1 in LUAD development in this work.

Based on this, we presented the expression level of RNH1 in the clinical samples form LUAD patients. Two LUAD cell lines NCI-H1395 and NCI-H1437 were used for in vitro experiments. The roles of RNH1 in cell proliferation, apoptosis, migration and invasion were investigated in vitro by overexpression and RNA-interference. Besides, the transcriptional regulation between E2F1 and RNH1 was explored by dual luciferase assay, ChIP detection, and pull-down assay. We further showed the differences in malignant behavior of tumor cells after co-overexpression of RNH1 and E2F1. In vivo experiments confirmed the roles of RNH1 in LUAD progression. High-throughput sequencing was performed to investigated the effect of RNH1 overexpression on the gene expression profile of LUAD cells. Our study may provide a theoretical basis for the treatment of LUAD.

## Materials and methods

### Clinical samples

Clinical and information was collected in the First Affiliated Hospital of China Medical University in keeping with institutional, national and Helsinki Declaration guidelines. All patients signed a written informed consent. Forty LUAD tissues and adjacent tissues without chemoradiotherapy were collected. The expression level of RNH1 was detected in 20 cancer and adjacent samples randomly selected out from the 40 samples. The paraffin section samples of 120 LUAD tissues were collected and immunohistochemical (IHC) staining was performed to detect the expression of RNH1 in the central location of the cancer tissue, and the correlation between the expression of RNH1 and the clinicopathological indicators of the tumor was analyzed. IHC scoring criteria refer to previous studies [8]. According to the percentage of positive cells, the immunopositivity was divided into 4 categories: <5% (score = 0), 5-10% (score = 1), 10-50% (score = 2), and ≧ 50% (score = 3). Staining intensity score ranged from 0 to 3, negative staining; 1, weak staining; 2, moderate staining; and 3, strong staining. The intensity and percentage scores are added to give a single immunohistochemical score from 0 to 6. Patients were divided into two groups: low RNH1 expression (score < 4) and high RNH1 expression (score ≥ 4). This study was approved by the Ethics Committee of the First Affiliated Hospital of China Medical University.

### Cell culture and treatment

NCI-H1975 cells, NCI-H1395 cells, NCI-H1437 cells and HCC827 cells were cultured in RPMI-1640 medium (Solarbio, Beijing, China) containing 10% fetal bovine serum (Tianhang, Zhejiang, China). A549 cells were cultured in F-12K medium (Servicebio, Wuhan, China) containing 10% fetal bovine serum. BEAS-2B cells were cultured in DMEM medium (Servicebio, China) containing 10% fetal bovine serum. They were cultured at 37℃ in a 5% CO_2_ incubator (Healforce, Shanghai, China). A549 cells were purchased from Prilosec and all other cells were purchased from icellbioscience. Based on the experimental results, two LUAD cell lines with intermediate RNH1 expression were selected for subsequent experiments. RNH1-specific shRNA was designed according to the gene coding sequence (NM_002939.4) and synthesized by Generalbiol (Anhui, China). The cells were divided into four groups: mock, shNC, shRNH1-1 and shRNH1-2. ShRNH1-1 sequence was: 5’-GCGGTGTGACATCAGACAACT-3’. ShRNH1-2 sequence was: 5’-GCTCTGCACTTCGAGTCAACC-3’. The single-stranded shRNA was then annealed to produce a double-stranded shRNA. Subsequently, shRNA was cloned into the vector pLKO.1-EGFP-puro (Fenghbio, Hunan, China; ZT101), and the restriction site was AgeI/Eco. A RNH1 overexpression plasmid (oe-RNH1) and an empty plasmid (vector-1) were constructed to overexpress RNH1. The lentivirus was infected into cells, which were divided into three groups: mock, vector-1 and oe-RNH1. Cells were divided into two groups (vector-2, oe-E2F1) and transfected with an empty plasmid (vector-2) or overexpression plasmid (oe-E2F1).

### Animal experiments

This study was approved by Laboratory Animal Welfare and Ethics Committee of China Medical University. Six-week-old male BALB/c nude mice, purchased from Changzhou Cavins Laboratory Animal Co., Ltd, were fed at a controlled temperature of 24 ± 1 °C and 45–55% humidity with free access to food and water. Experiments were started one week later. NCI-H1395 cells (5 × 10^6^) at logarithmic growth stage were injected subcutaneously into the right axilla of mice. After growing to a tumor visible to the naked eye, the tumor volume was measured every 3 days. Mice were executed on the 24th day after injection, and the tumors were removed and weighed. Carbon dioxide inhalation (a fill rate of 60% of the chamber volume per minute) was used for mouse euthanasia.

### MTT assay

According to the supplier’s instructions for the MTT kit from Beyotime (Shanghai, China), 10 µL MTT was added to each well and incubated at 37 °C for 4 h. Then 100 µL Formazan was added until fully dissolved, the OD value at 570 nm was measured using a microplate reader (Biotek, VT, USA).

### qRT-PCR

Total RNA was isolated from cells and tissues, then reverse-transcribed to cDNA using BeyoRT II M-MLV reverse transcriptase (Beyotime). Next, SYBR Green (Solarbio) was used to amplify cDNA. Glyceraldehyde 3-phosphate dehydrogenase (GAPDH) was used as the internal control to normalize data. The relative gene expression of mRNAs was determined by the 2^−ΔΔCt^ method. Primer sequences: E2F1-Forward: ACTCCTCGCAGATCGTCAT, E2F1-Reverse: TCCAGCCTCCGCTTCAC; RNH1-Forward: CCTGGGCAGCAACAAGC, RNH1-Reverse: AGTCGGGCACCCTCATC.

### Western blot

Cells and tissues were lysed in RIPA buffer (Solarbio) containing protease and phosphatase inhibitors. BCA Protein Assay kit (Beyotime) was used to measure the concentration of protein. Protein samples were separated by SDS-PAGE at 80 V for 2.5 h and then transfected into PVDF membranes (Millipore, MA, USA) for 2 h. After blocking in 5% fat-free milk for 1.5 h, the membranes were incubated with primary antibodies at 4℃ overnight (see Table. S1 for primary antibodies information). Next day, the membranes were incubated with secondary antibodies for 2 h: goat anti-rabbit IgG-HRP (Solarbio; SE134; 1:3000); goat anti-mouse IgG-HRP (Solarbio; SE131; 1:3000). Finally, the membranes were washed in TBST and detected by ECL chemiluminescent reagent (Solarbio).

### Clone formation

Each group of cells was inoculated in a culture dish. When naked-eye clones were formed, cells were wash twice with PBS, 4% paraformaldehyde (Aladdin, Shanghai, China) was used to fix the cells for 25 min. Rickey’s composite dye (Keygen, Nanjing, China) was used to stain the cells for 5 min. A general light microscope (Olympus, Tokyo, Japan) was used to count clones with more than 50 cells. Plate clone formation efficiency = (number of clones / number of cells inoculated) × 100%.

### Flow cytometry analysis

Cell cycle and apoptosis were determined by flow cytometry. Cells were collected and fixed overnight in 70% methanol at 4 °C. PBS was used to wash the cells twice, and then the cells were stained with 500 µL PI/RNase A (1:9 volume mix, Keygen) for 30 min at room temperature in darkness. The stained cells were characterized by flow cytometry (Agilent, CA, USA), and FlowJo software was used to analyze the cell cycle. Cells were collected by centrifugation at 150 g for 5 min, and the supernatant was discarded. Annexin V-Light 650 (Wanleibio, Shengyang, China) was added and mixed, and then 5 µL PI (Keygen) staining solution was added. After incubation for 15 min at room temperature, apoptosis was detected on a flow cytometer.

### Hoechst stain

Cells were washed twice with PBS and then fixed for 20 min at room temperature with 0.5 mL of fixative. Then 0.5 mL of Hoechst staining solution (Keygen) was added, mixed and stained for 5 min. Anti-fluorescence quenching blocking solution was dropped on the slide. Cells were then observed and photographed under a fluorescence microscope (Olympus).

### IHC staining

After deparaffinization and dehydration, the paraffin sections were boiled in sodium citrate solution (0.1 M) for 15 min. Next, endogenous peroxidase activity was blocked with 3% hydrogen peroxide (Sinoreagent, Shanghai, China). Sections were incubated with PCNA antibody, MMP9 antibody or Cleaved caspase 3 antibodies at 4℃ overnight. The next day, sections were incubated with a secondary antibody (HRP-labeled goat anti-rabbit IgG; thermoFisher, PA, USA; #31,460; 1:500) for 1 h at room temperature. The staining was observed under the microscope (Olympus).

### Terminal deoxynucleotidyl transferase-mediated dUTP nick end labeling (TUNEL) staining

Sections were fixed in 4% paraformaldehyde for 25 min at 4 °C, washed twice in PBS and then treated with 0.1% Triton X-100 (Beyotime) for 5 min. Sections were then incubated in TUNEL working fluid (Enzyme solution: Label solution, 1:9). DAPI (Aladdin) was used to stain nuclei, and fluorescent quenchers (Solarbio) were dropped to seal the slides. The sections were observed under a microscope and photographed (Olympus).

### Transwell invasion and migration assays

For invasion assays, Transwell chambers (Labselect, Hefei, China) containing matrigel (Corning, NY, USA) were placed in 24-well plates, and 700 µL culture medium containing 10% FBS was added to the lower chamber. Cell suspension was added into the upper chamber. For the migration assay, the Transwell chamber was placed in a 24-well plate, and 700 µL culture medium containing 10% FBS was added to the lower chamber. Cell suspension was added to the upper chamber and cultured in a 5% CO2 incubator at 37℃. The Transwell chamber was washed twice with PBS, fixed with 4% paraformaldehyde for 20 min at room temperature, and stained with 0.5% crystal violet (Amresco, Shanghai, China) for 5 min. The cells in the lower layer of the microporous membrane were counted under an inverted microscope (Olympus), and 5 fields were selected for each sample to count the number of cells.

### Enzyme linked immunosorbent assay (ELISA)

The levels of matrix metalloproteinase2 (MMP2) and matrix metalloproteinase9 (MMP9) in the cell supernatant were determined by ELISA kit (Liankebio, Hangzhou, China). The samples were sequentially added to the microwells coated with monoclonal antibodies and then incubated with HRP-labeled antibodies. Next, substrate was added for color development. Absorbance at 450 nm wavelength was measured with a microplate reader (Biotek).

### Dual luciferase assay

Dual luciferase assay was used to detect whether E2F1 binds to the different segment or group of the RNH1 promoter (-89 ∼ -82; -1064 ∼ -1054; -1514 ∼ -1504). The cell culture medium was aspirated, cells were washed with PBS, and 250 µL cell lysate was added. Then, 100 µL of firefly luciferase reagent (Keygen) was added to each well, and 20 µL cell sample was added. Next, 100 µL Renilla luciferase reagent (Keygen) was added to each well. Luciferase activities were detected using multifunctional microplate reader (Biotek). The transfection efficiency was calculated with the ratio of firefly luciferase/Renilla luciferase activity.

### Chromatin immunoprecipitation (ChIP) assay

ChIP detection was performed using a ChIP-IT kit (Wanleibio). Briefly, cells were fixed with formaldehyde and then dissolved. To precipitate DNA fragments, 2 µg of anti-E2F1 antibody or normal IgG was used. After immunoprecipitation, protein DNA cross-linking was reversed, DNA was purified and then amplified by 2×Power Taq PCR MasterMix (Biotek) with RNH1 promoter-specific primers. Based on the results of dual luciferase assay, the primers of RNH1 were designed and the sequences of primer were as following: primer 1 (Forward GCTCAGAACACTCGGGACG, Reverse CGACTCTAGGCGACCTTGGT); primer 2 (Forward TGAGCCCAATTCAAGACC, Reverse CTCCACTGCTTAGGCTGTAC). qRT-PCR products were detected by DNA electrophoresis and visualized by ethidium bromide staining.

### Pull-down assay

GST pull-down assays were performed according to the manufacturer’s instructions of DNA pulldown assays kit. Cells expressing the given GST fusion protein were harvested by centrifugation at 1000 g for 5 min at 4 °C. Cells were lysed in RIPA buffer supplemented with protease inhibitor and DTT on ice to extract nucleoproteins. Protein samples were removed with DNase, DNase salt stock and Agarose beads to remove nucleic acids and pre-washed. A double-stranded oligonucleotide probe consisting of RNH1 promoter region sequences was synthesized and used. The protein samples were added to the probe-magnetic bead complexes and incubated for 1 h at 4 °C after rotational binding. After repeated washing, the beads were collected and the supernatant was removed. The magnetic beads were eluted with Protein Elution buffer and DTT, and incubated at 37℃ for 2 h. Next, the magnetic beads were collected and the supernatant was extracted. Finally, the removed proteins were detected by Western blot.

### Bioinformatics analysis

RNH1 was overexpressed in NCI-H1395 and NCI-H1437 cells, and cell samples were collected and sent to Hangzhou Lianchuan Biotechnology Co., LTD (China). High-throughput mRNA sequencing was performed on NCI-H1395 and NCI-H1437 cells overexpressing RNH1, and the effective sequencing results were integrated and extracted. After confirming the quality of samples, principal component analysis (PCA) was performed. R statistical programming language was used to analyze the differentially expressed genes among samples. The up-regulated and down-regulated genes in NCI-H1395 and NCI-H1437 cells were intersected. KEGG analysis were performed for the high and low expression genes selected after intersection. The screening criteria of differential genes were │log2FoldChange│> 1 and P value < 0.05.

### Statistical analysis

Statistical analyses were performed with GraphPad Prism 8.0. Data were presented as mean ± SD. Comparisons between two groups were done by t-test, comparisons between multi-group were done by one-way ANOVA. When there were different times and different processing effects, two-way ANOVA was used. Statistical significance was set at *P* < 0.05. All experiments were performed at least three times unless otherwise stated.

## Results

### RNH1 expression was low in LUAD tissues of patients

**Fig. 1:**
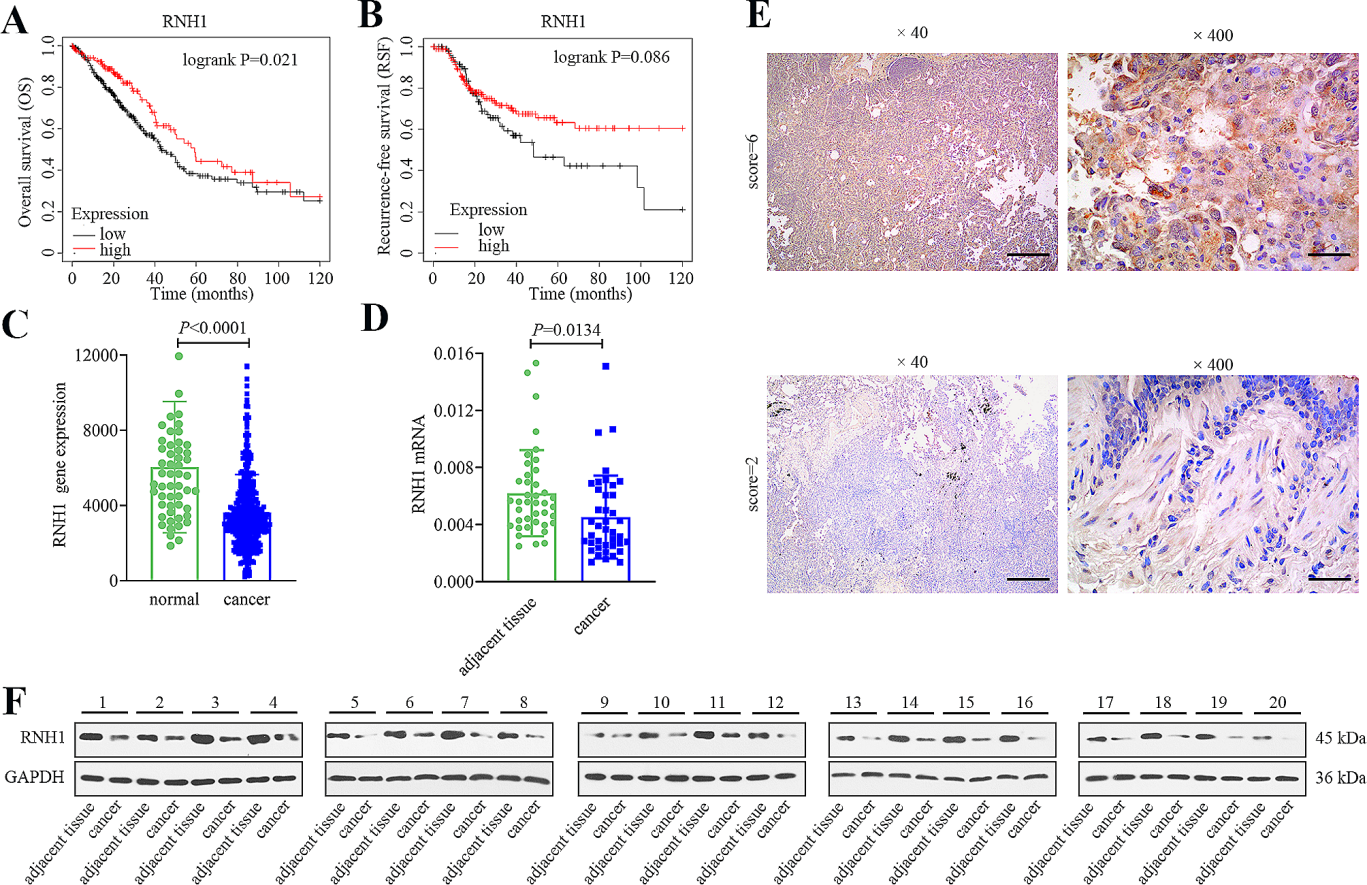
RNH1 expression was low in LUAD tissues of patients. (**A**) Overall survival and (**B**) recurrence-free survival of LUAD patients with low and high RNH1 levels from the Kaplan-Meier analysis. (**C**) The RNH1 gene expression between LUAD tissues and normal tissues were compared by TNM plot (https://www.tnmplot.com/) (normal: 53 samples, tumor: 465 samples). (**D**) RNH1 mRNA level in 40 cases of LUAD tissues and adjacent tissues without chemoradiotherapy. (**E**) Typical results of IHC staining from two patients. The scores were 6 and 2, respectively. 40× magnification, scale bar: 500 μm; 400× magnification, scale bar: 50 μm. (**F**) Western blot was performed to detect the protein levels of RNH1 in 20 LUAD patients. Full-length blots were presented in Supplementary data. *P* < 0.05 was considered statistically significant

The clinical significance of RNH1 in LUAD was studied by database analysis (http://kmplot.com). Kaplan-Meier curve of LUAD patients showed that high RNH1 levels indicated an increased overall survival of LUAD (Fig. 1A), and there was a trend toward improved recurrence-free survival among patients with high RNH1 expression (Fig. 1B). TNM plot also showed the low expression of RNH1 in LUAD tissues (Fig. 1C). Besides, we determined mRNA expression of RNH1 in 40 pairs LUAD cancer tissues and adjacent normal tissues. The results showed that mRNA expression of RNH1 was reduced in tumor tissue (Fig. 1D). RNH1 protein levels were lower in cancer tissue than in adjacent tissue in all 20 LUAD patients (Fig. 1F). Figure 1E illustrated the representative images of RNH1 IHC staining and scores of two typical patients. The IHC scores were 6 (strongly positive) and 2 (negative), respectively. We then assessed the correlation between RNH1 expression and patients’ clinicopathological features. As shown in Table. S2, RNH1 expression was significantly correlated with primary tumor, lymph node metastasis, clinical stages and TNM stages.

### RNH1 inhibited cell proliferation of LUAD

**Fig. 2:**
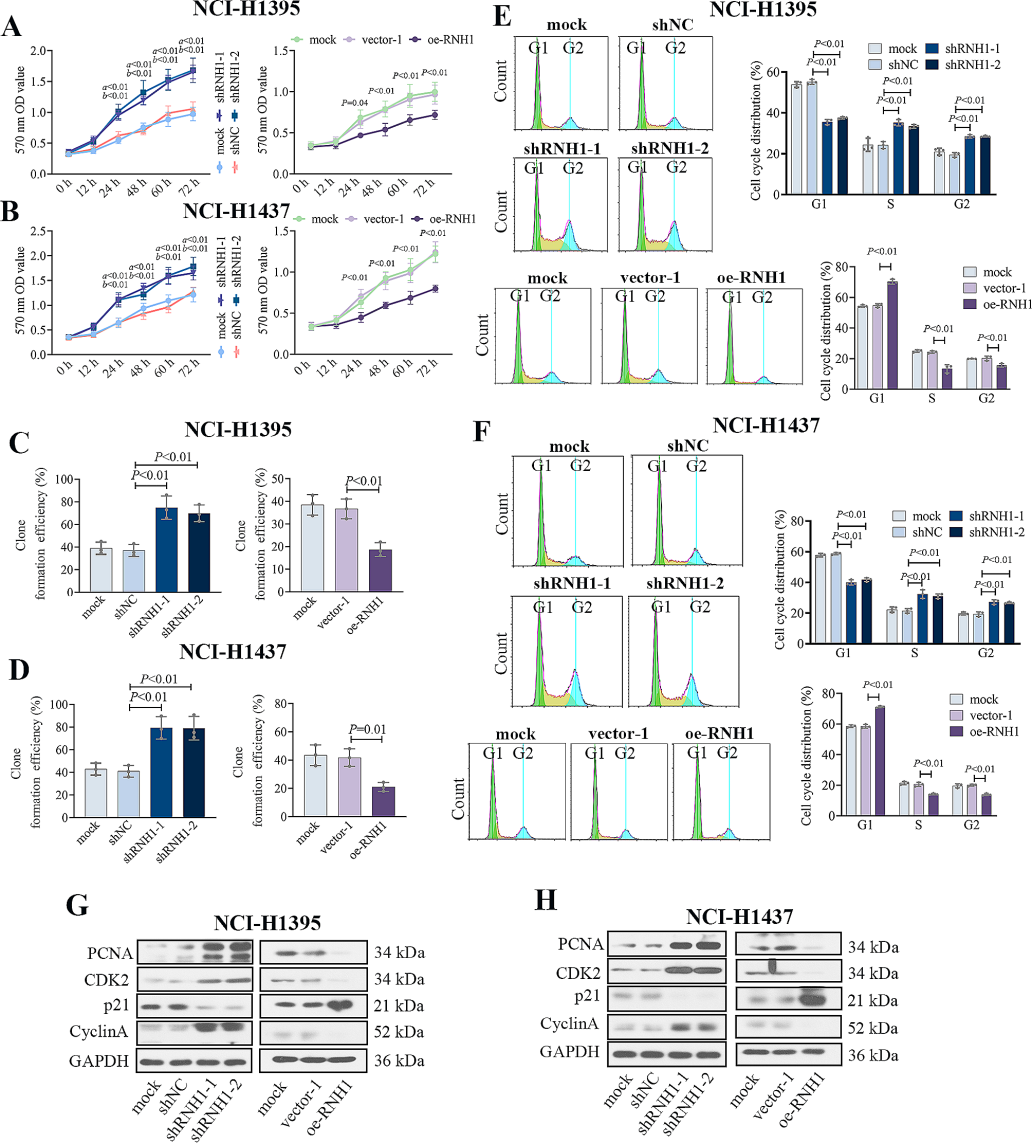
RNH1 inhibited cell proliferation of LUAD. MTT assay was performed to detect the proliferation ability of NCI-H1395 cells (**A**) and NCI-H1437 cells (**B**). Colony formation assays were utilized to evaluate proliferative capacity of NCI-H1395 cells (**C**) and NCI-H1437 cells (**D**). Flow cytometry was used to assess the cycle distribution of NCI-H1395 cells (**E**) and NCI-H1437 cells (**F**). Western blot was performed to detect the expression of PCNA, CDK2, p21 and cyclin A in NCI-H1395 cells (**G**) and NCI-H1437 cells (**H**)

In this part, we explored the roles of RNH1 in LUAD cell lines by RNH1 overexpression or silencing. We verified the expression of RNH1 in LUAD cell lines (NCI-H1975, NCI-H1395, A549, NCI-H1437, HCC827) and human normal lung epithelial cells (BEAS-2B) by western blot. The expression of RNH1 in NCI-H1395 and NCI-H1437 cells was intermediate, so NCI-H1395 and NCI-H1437 cells were selected for subsequent experiments (Supplementary Fig. 1A). The expression of RNH1 was decreased in NCI-H1395 and NCI-H1437 cells when RNH1 was knocked down, RNH1 expression was increased when RNH1 was overexpressed (Supplementary Fig. 1B and C). Cell proliferation assays showed that cell lines transfected with shRNH1 demonstrated significant increase in proliferation after 24 h, and the proliferation ability of LUAD cells decreased after 24 h when RNH1 was overexpressed (Fig. 2A-B). This suggested that RNH1 might inhibited the proliferation ability of LUAD cells. The clone formation results supported it with similar results (Fig. 2C-D). Cell cycle of NCI-H1395 and NCI-H1437 cells were detected by flow cytometry (Fig. 2E-F). Knockdown of RNH1 decreased the proportion of cells in the G1-phase of the cell cycle, increased the proportion of cells in S-phase and G2-phase. RNH1 overexpression produced the opposite results. Besides, cell cycle-related protein expression of PCNA, CDK2 and Cyclin A was increased after RNH1 knockdown, and p21 expression was decreased. Overexpression of RNH1 reduced the protein expression of PCNA, CDK2 and Cyclin A and increased the expression of p21 (Fig. 2G-H). Collectively, RNH1 inhibited LUAD cell proliferation.

### RNH1 promoted the apoptosis of LUAD cell

**Fig. 3:**
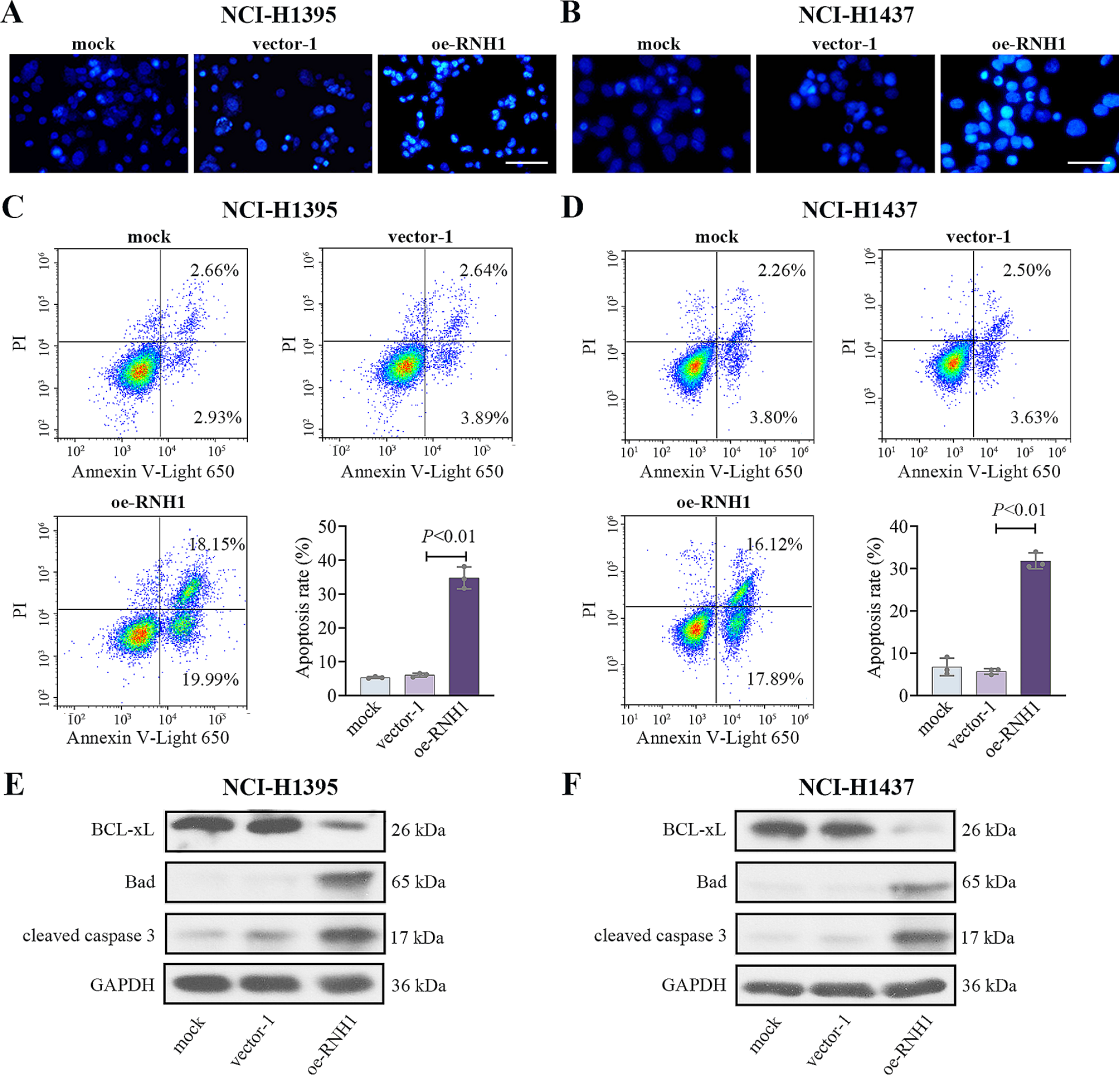
RNH1 promoted cell apoptosis of LUAD. Hoechst 33,342 staining was used to assess morphological changes in apoptosis in NCI-H1395 cells (**A**) and NCI-H1437 cells (**B**). Scale bar, 50 μm. Flow cytometry was used to determine apoptosis in NCI-H1395 cells (**C**) and NCI-H1437 cells (**D**). Western blot was performed to detect the levels of BCL-xL, Bad, and cleaved caspase 3 in NCI-H1395 cells (**E**) and NCI-H1437 cells (**F**). Full-length blots were presented in Supplementary data. shNC, negative control; vector-1, empty vector 1. *N* = 3. *P* < 0.05 was considered statistically significant

Moreover, the effects of RNH1 on cell apoptosis of LUAD was determined by Hoechst33342 (blue) staining and Flow cytometry. Nuclear were visualized by Hoechst33342 (blue) staining in NCI-H1395 and NCI-H1437 cells (Fig. 3A-B). Compared with the cells treated with vector-1, brighter blue fluorescence nucleus with a high condensed chromatin and nuclei shrinkage was observed in RNH1-overexpreseed cells, which suggested an obvious induction of cell apoptosis caused by RNH1. Results of flow cytometry also showed that RNH1 overexpression led to an increase in the number of apoptotic cells, and the apoptotic rate of cells was increased both in NCI-H1395 and NCI-H1437 cells (Fig. 3C-D). Besides, the expression of the anti-apoptotic protein Bcl-xL was decreased and the levels of the pro-apoptotic proteins Bad and cleaved caspase 3 were increased (Fig. 3E-F). Collectively, RNH1 might promote LUAD cell apoptosis.

### RNH1 inhibited LUAD cell migration and invasion

**Fig. 4:**
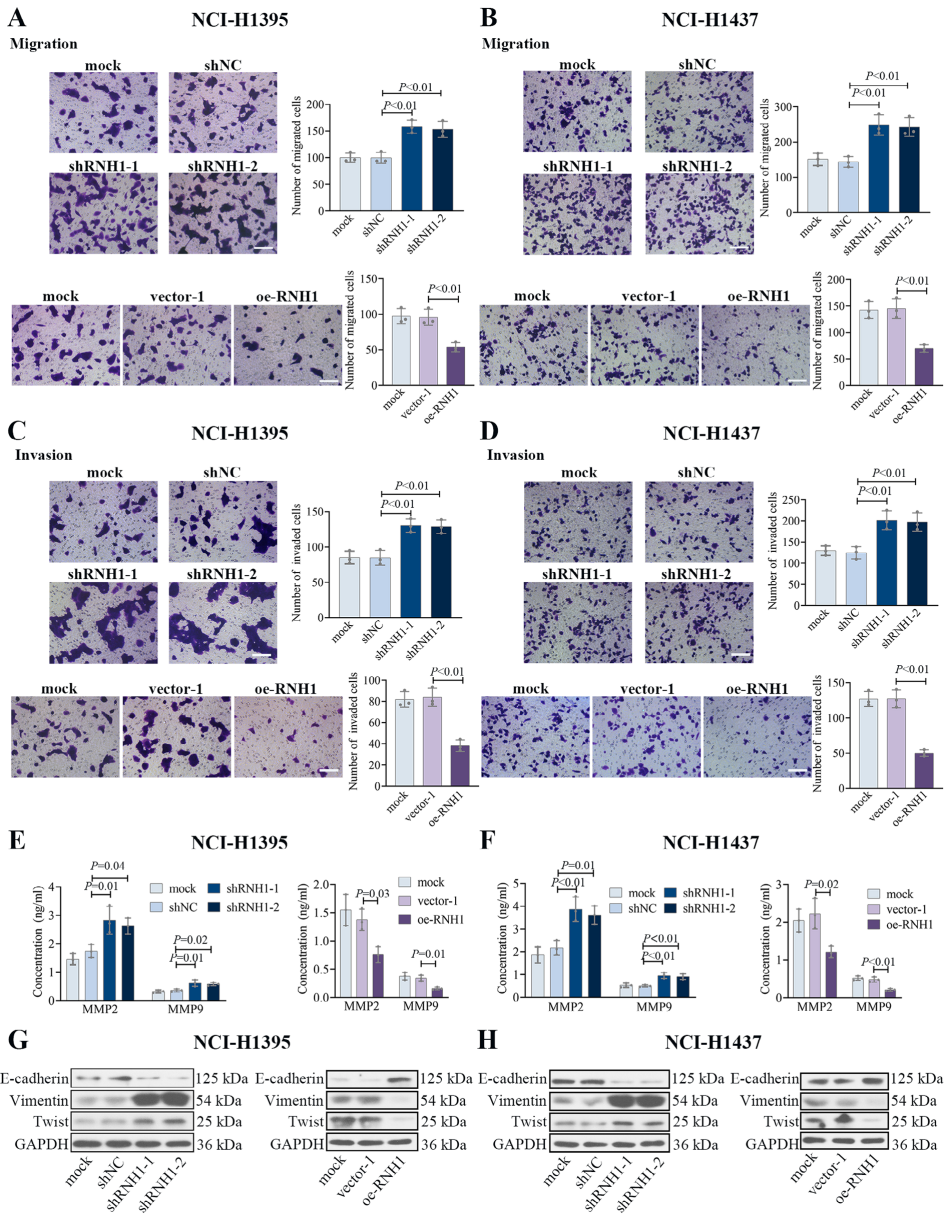
RNH1 inhibited LUAD cell migration and invasion. The migration ability of NCI-H1395 cells (**A**) and NCI-H1437 cells (**B**) was evaluated by Transwell assay. The invasion ability of NCI-H1395 cells (**C**) and NCI-H1437 cells (**D**) was evaluated by Transwell assay. Scale bar, 100 μm. The expression of MMP2 and MMP9 in NCI-H1395 cell supernatant (**E**) and NCI-H1437 (**F**) cell supernatant was detected by ELISA. The expression of E-cadherin, Vimentin and Twist in NCI-H1395 cells (**G**) and NCI-H1437 cells (**H**) were determined by Western blot. Full-length blots were presented in Supplementary data. shNC, negative control; vector-1, empty vector 1. *N* = 3. *P* < 0.05 was considered statistically significant

The migration and invasion ability of LUAD cells were evaluated by Transwell assay. As shown in Fig. 4A-B, in NCI-H1395 and NCI-H1437 cells, the number of migrated cells were increased after RNH1 knockdown and RNH1 overexpression decreased the number of migrated cells. For invasion ability, the results were approximately similar to migration results (Fig. 4C-D), which might indicate that RNH1 inhibited the migration and invasion ability of NCI-H1395 and NCI-H1437 cells. In addition, the concentration of MMP2 and MMP9 in cell supernatants was detected by ELISA. MMP9 and MMP2 are common indicators of cancer migration and invasion. RNH1 knockdown increased the intracellular MMP2 and MMP9 levels of NCI-H1395 and NCI-H1437 cells, whereas the results were reversed when RNH1 was overexpressed (Fig. 4E-F). Key epithelial–mesenchymal transition (EMT) characteristics including E-cadherin, Vimentin and Twist were assessed by western blot. It was indicated that RNH1 knockdown decreased the protein levels of E-cadherin and increased the protein levels of Vimentin and Twist, whereas overexpression of RNH1 produced the opposite results (Fig. 4G-H). These all suggested that RNH1 inhibited LUAD cell migration and invasion.

### RNH1 expression was negatively regulated by E2F1 transcription factor

**Fig. 5:**
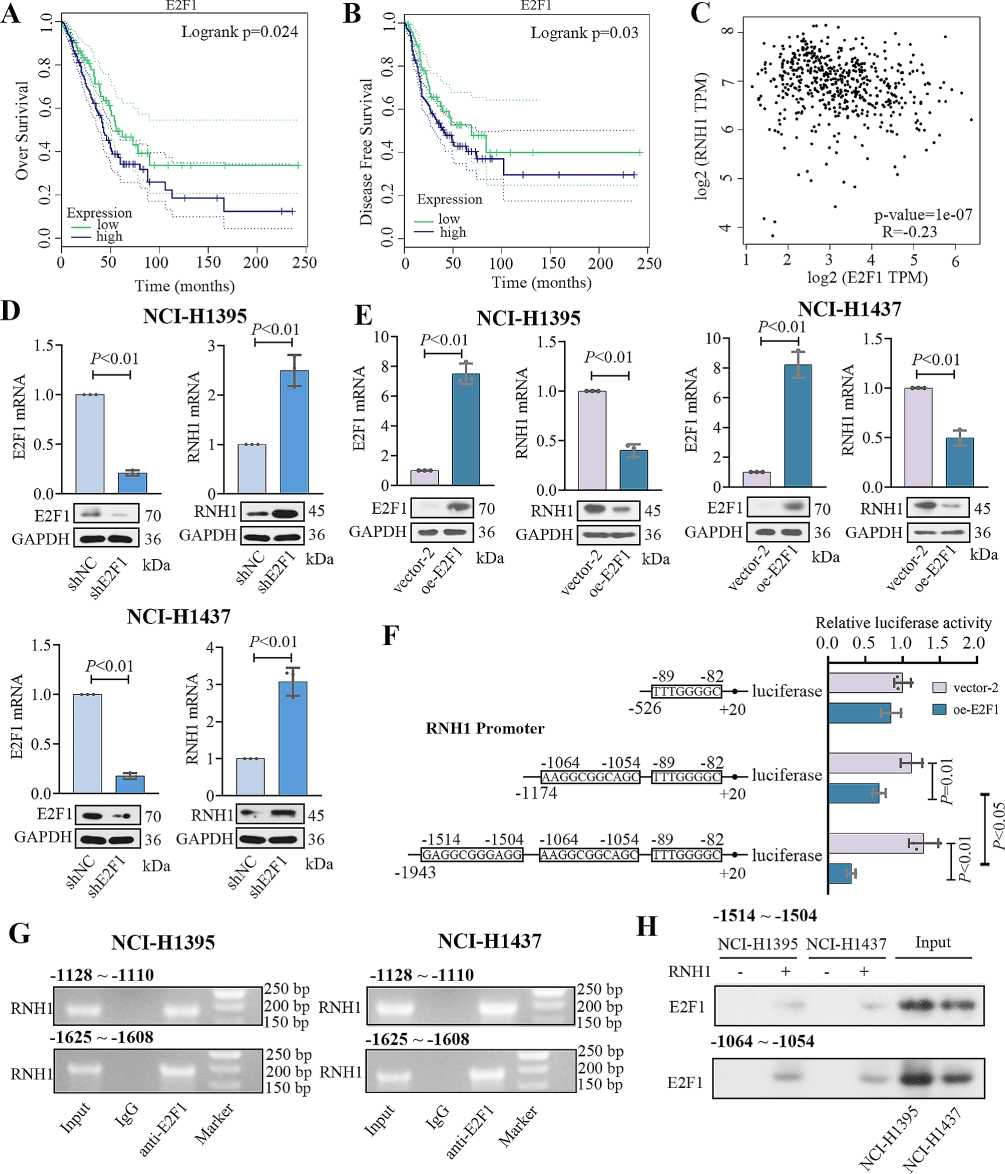
RNH1 expression was negatively regulated by E2F1 transcription factor. (**A**) Overall survival and (**B**) disease-free survival of LUAD patients with low and high E2F1 levels from GEPIA database. (**C**) The correlation between E2F1 and RNH1 (GEPIA). (**D-E**) qRT-PCR and Western blot were used to detect the levels of E2F1 and RNH1 in NCI-H1395 cells and NCI-H1437 cells. Full-length blots were presented in Supplementary data. (**F**) Dual luciferase assay was used to detect possible E2F1 binding sites in the RNH1 promoter. (**G**) ChIP assay was performed in NCI-H1395 cells and NCI-H1437 cells using anti-E2F1 antibody against RNH1 promoter primers. (**H**) The pull-down assay was used to determine the interaction between E2F1 and RNH1 in NCI-H1395 cells and NCI-H1437 cells. vector-2, empty vector 2. *N* = 3. *P* < 0.05 was considered statistically significant

Previous studies indicated that E2F1 acted as a tumor-promoting factor. GEPIA database showed that high E2F1 expression indicated a poorer overall survival and disease-free survival time (Fig. 5A-B). Interestingly, correlation analysis form GEPIA presented a negative correlation between the E2F1 expression and RNH1 expression (Fig. 5C). Next, the regulation between RNH1 and E2F1 was further investigated in LUAD cell lines by E2F1 overexpression and knockdown. qRT-PCR and western blot were used to detect the overexpression and knockdown efficiency of E2F1 and the level of RNH1 in cells (Fig. 5D-E). The expression of RNH1 was increased when E2F1 was silenced, while E2F1 overexpression decreased RNH1 expression. According to the jasper database, E2F1 can bind to the promoter of RNH1. Dual luciferase reporter assay, ChIP assay, and pull-down assay were then performed to confirm this binding relationship. Two promoter sequences of RNH1 were inserted into the reporter gene vector, respectively. E2F1 bound to the RNH1 promoter (-1064 ∼ -1054, -1514 ∼ -1504) and reduced its transcriptional activity (Fig. 5F). ChIP and pull-down assay were carried out in NCI-H1395 and NCI-H1437 cells. We found that E2F1 was significantly enriched at the RNH1 promoter (-1148 ∼ -943, -1628 ∼ -1423) (Fig. 5G). The E2F1 protein pulled down by the biotin-labeled RNH1 probe increased in transformed cells (Fig. 5H). These results demonstrated that RNH1 expression might be negatively regulated by E2F1.

### The roles of RNH1 in LUAD were regulated by E2F1

**Fig. 6:**
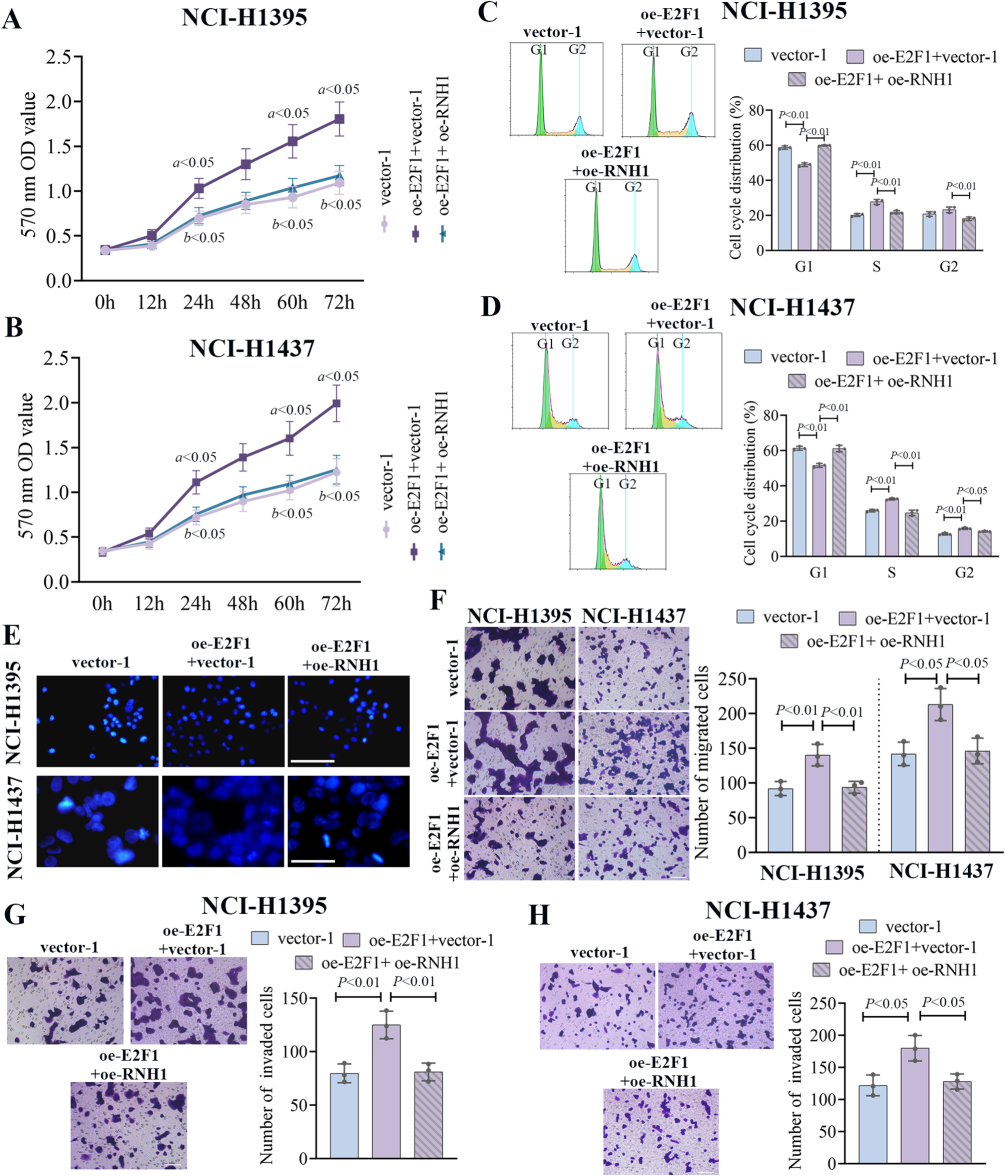
The roles of RNH1 in LUAD were regulated by E2F1. MTT assay was performed to detect the proliferation ability of NCI-H1395 cells (**A**) and NCI-H1437 cells (**B**). Flow cytometry was used to assess the cycle distribution of NCI-H1395 cells (**C**) and NCI-H1437 cells (**D**). (**E**) Hoechst 33,342 staining was used to assess morphological changes in apoptosis in NCI-H1395 cells and NCI-H1437 cells. Scale bar, 50 μm. (**F**) The migration ability of NCI-H1395 cells and NCI-H1437 cells. Scale bar, 100 μm. The invasion ability of NCI-H1395 cells (**G**) and NCI-H1437 cells (**H**). Scale bar, 100 μm. *N* = 3. a, oe-E2F1 + vector-2 vs. vector-2; b, oe-E2F1 + oe-RNH1 vs. oe-E2F1 + vector-2. a, b and *P* < 0.05 was considered statistically significant

To further confirm the regulation between E2F1 and RNH1, two LUAD cell lines were co-transfected with E2F1-overexpressing plasmid and RNH1-overexpressing plasmid. In the cell proliferation experiment using MTT assay, E2F1 enhanced the cell proliferation of LUAD, but RNH1 overexpression reversed it (Fig. 6A-B). Besides, cell cycle distribution showed that E2F1 accelerated the G1-S transition while RNH1 arrested it (Fig. 6C-D). Hoechst assay indicated that E2F1 overexpression inhibited the cell apoptosis, and it was reversed after RNH1 overexpression (Fig. 6E). Moreover, the regulation between E2F1 and RNH1 on cell migration and invasion was assessed. As shown in Fig. 6F, more migrated cells were observed in cells with E2F1 overexpression alone, while it was offset by RNH1 overexpression. Consistently, E2F1 overexpression alone increased the number of invaded cells (Fig. 6G). By contrast, a significant decrease was observed in cells with co-transfection of E2F1 overexpression plasmid and RNH1 overexpression plasmid (Fig. 6H). These findings further delineated the mechanism of action of E2F1 in RNH1 mediated cancer progression in LUAD cell lines.

### RNH1 inhibited LUAD tumorigenesis in vivo

**Fig. 7:**
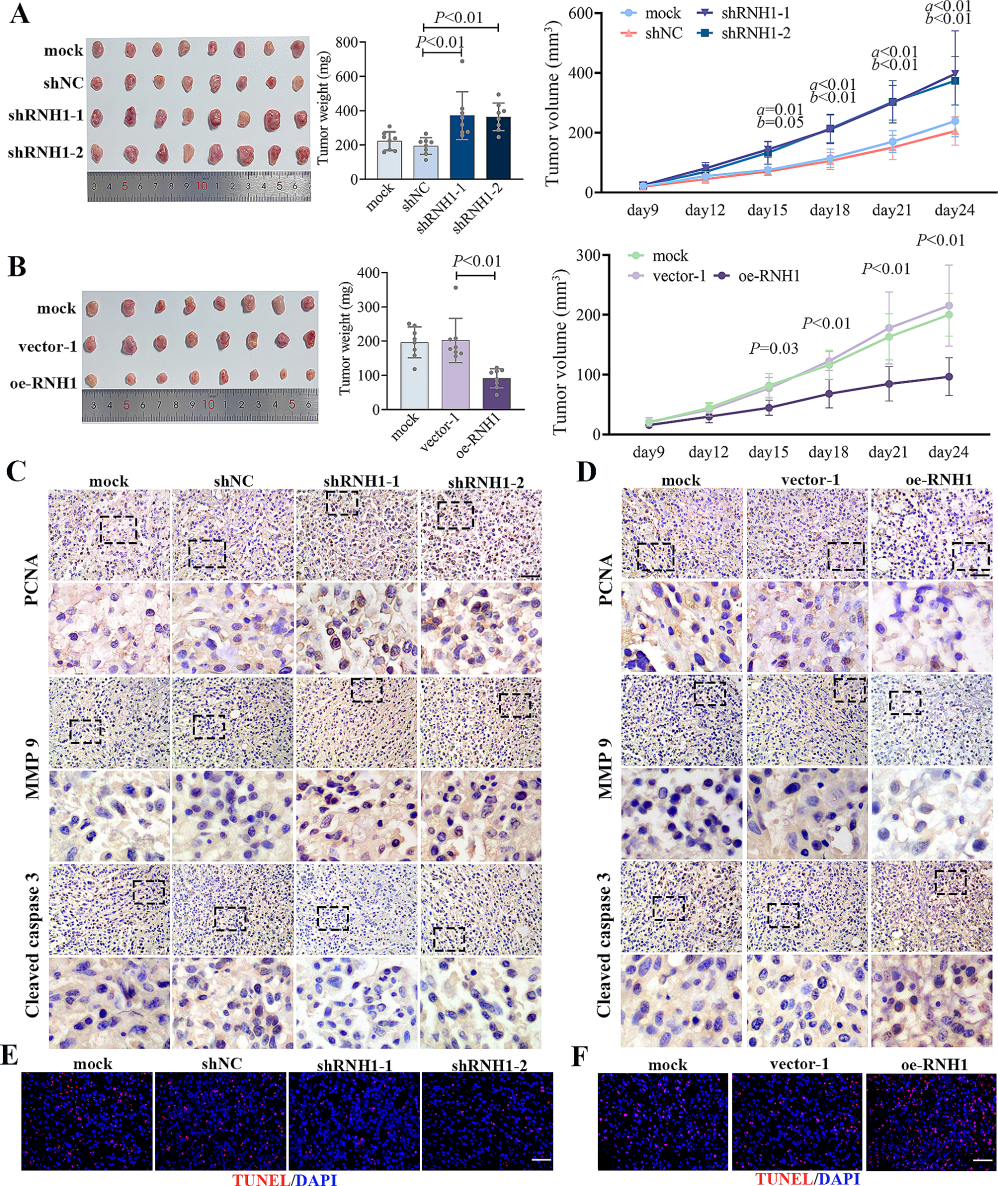
RNH1 inhibited LUAD tumorigenesis in vivo. (**A-B**) The nude mice were sacrificed on the 24th day after the injection of LUAD tumor cells (5 × 10^6^), and the tumor tissues were removed, photographed, measured and weighed. (**C-D**) IHC detection of PCNA, MMP9, and Cleaved caspase 3 expression in tumor tissues. Scale bar, 50 μm. (**E-F**) TUNEL staining was performed to measure apoptosis in tumor tissues. Scale bar, 50 μm. *N* = 8. vector-1, empty vector 1. a, shRNH1-1 vs. shNC; b, shRNH1-2 vs. shNC. a, b and *P* < 0.05 was considered statistically significant

The tumor formation model was performed to illustrate the effects of RNH1 on tumor development in vivo. We injected NCI-H1395 cells with stable RNH1 knockdown or overexpression into the armpit of nude mice. The tumor weight was increased when RNH1 was knocked down. When RNH1 was overexpressed, tumor weight was decreased. The volume of the tumor on days 15 to 24 also reflected this phenomenon (Fig. 7A-B). In addition, IHC staining was performed to detect the expression of PCNA, MMP9, and cleaved caspase 3 in tumor tissues (Fig. 7C-D). RNH1 knockdown upregulated the expression of PCNA and MMP9, and RNH1 overexpression declined it, suggesting that RNH1 inhibited LUAD tumor formation in vivo. Besides, it was also found that the expression of cleaved caspase 3 was significant upregulated by RNH1 overexpression. We then assessed cell apoptosis in tumor tissues by TUNEL (red) staining. As shown in Fig. 7E-F, RNH1 knockdown decreased apoptosis of LUAD cells, while RNH1 overexpression had the opposite effects, which was consistent with the above in vitro experimental results. Together, RNH1 exerted suppressive effects on tumorigenesis of LUAD in vivo.

### Overexpression of RNH1 resulted in changes in mRNA expression profile of LUAD cells

**Fig. 8:**
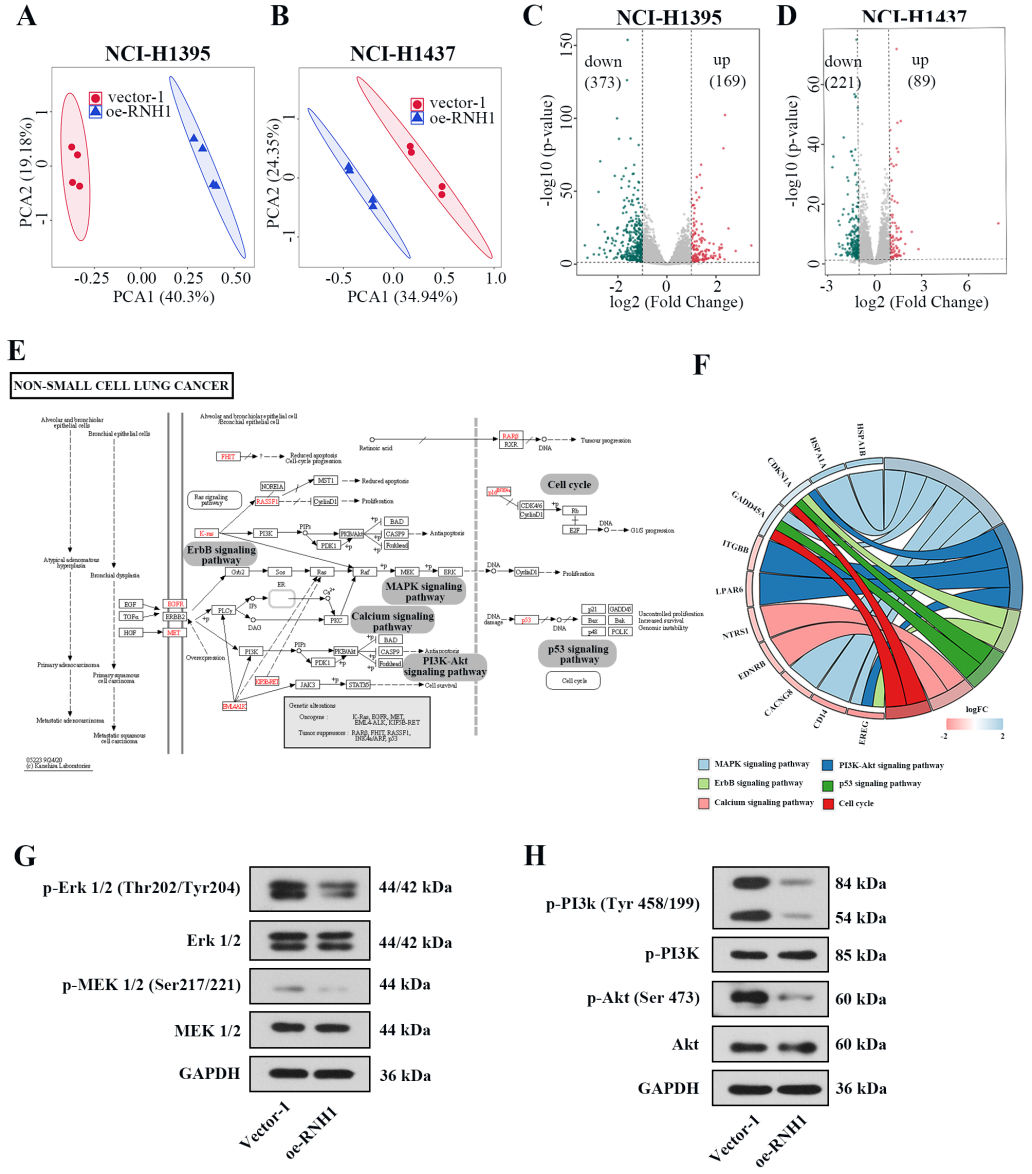
Overexpression of RNH1 resulted in changes in mRNA expression profile of LUAD cells. Principal component analysis (PCA) of samples in NCI-H1395 cells (**A**) and NCI-H1437 cells (**B**). Volcano plots ofdifferential expression genes in NCI-H1395 cells (**C**) and NCI-H1437 cells (**D**). (**E**) The KEGG map of non-small cell lung cancer. (**F**) Chord diagram for KEGG pathways related to LUAD. (**G**) The protein level of p-Erk 1/2, Erk 1/2, p-MEK 1/2 and MEK 1/2 in NCI-H1395 cells. (**H**) The protein level of p-PI3K, PI3K, p-Akt and Akt in NCI-H1395 cells. *N* = 3

High-throughput mRNA sequencing was performed on NCI-H1395 and NCI-H1437 cells with RNH1 overexpression. Principal component analysis (PCA) revealed that the replicates were highly consistent and gene expression profile were clearly distinct (Fig. 8A-B). Volcanic map presented the differentially expressed genes with a threshold of│log2FoldChange│> 1 and *P* value < 0.05. In NCI-H1395 cells, 169 genes were up-regulated and 373 genes were down-regulated. In NCI-H1437 cells, 89 genes were up-regulated and 221 genes were down-regulated (Fig. 8C-D). Intersection analysis identified 43 up-regulated genes and 123 down-regulated genes in NCI-H1395 and NCI-H1437 cells (Fig.S2A-B). GO enrichment analysis (Fig.S2C-D) and KEGG analysis (Fig.S2E-F) were performed for the high and low expression genes selected after intersection and presented the top 20 enriched pathways. We also found that the differentially expressed genes were enriched in signaling pathways in LUAD, like MAPK signaling pathway, PI3K-Akt signaling pathway, ErbB signaling pathway, p53 signaling pathway, Calcium signaling pathway, and Cell cycle (Fig. 8E-F). In the follow-up experiments, we determined the effects of RNH1 on the activation of MAPK signaling pathway and PI3K-Akt signaling pathway in NCI-H1395 cells. As expected, the level of p-Erk 1/2 and p-MEK 1/2 was decreased by RNH1 overexpression (Fig. 8G). Contrast to the cells transfected with vector-1, lower level of p-PI3K and p-Akt was showed in RNH1-overexpressed cells (Fig. 8H). Besides, results described above indicated that RNH1 inhibited the protein level of CDK2 and p21 in LUAD cell lines. These results suggested that RNH1 inhibited the activation of these signaling pathways, thereby blocking tumor progression of LUAD.

## Discussion

Lung cancer is a major cancer killer and poses a serious challenge to patients and clinicians [24]. LUAD is the most common subtype of lung cancer and has a low survival rate [25]. In recent years, the occurrence, development and treatment of LUAD have been of interest to many medical researchers. The discovery of validated potential biomarkers is important to predict prognosis and immune response in patients with LUAD [26, 27]. MA Sarangdhar and R Allam [5] reviewed that the expression of RNH1 was reduced in cancer tissues, and overexpression of RNH1 in vitro weakened the ability of cell proliferation, migration and invasion. According to public database, RNH1 expression was reduced in LUAD tissues, and analysis of clinical samples in the present work validated this finding. In this study, RNH1 was successfully overexpressed or knocked down by constructing interference plasmids in two LUAD cell lines. We focus on the role of E2F1/RNH1 axis in LUAD and determine whether RNH1 is an effective therapeutic target for LUAD.

As a core transcription factor, E2F1 is a key factor in tumorigenesis and metastasis of LUAD [28]. Our study illustrated the importance of RNH1 in LUAD and the potential regulation between E2F1 and RNH1 in the process of tumor formation. Here, RNH1 expression was shown to be inversely correlated with E2F1. Further molecular experiments implied that E2F1 could bind to the RNH1 promoter and repress RNH1 expression. Previous study reported that transcriptional inhibition of E2F1 inhibits renal cell carcinoma progression [29]. Disruption of E2F1 stability inhibits the proliferation of LUAD cells [22]. Our results may reflect a negative regulatory mechanism between RNH1 and E2F1, which further reveal their function in LUAD.

Cell growth and proliferation involve an orderly progression of different cell cycle phases [30]. RNH1 has been reported as a potential biomarker for cholangiocarcinoma due to high autoantibody levels [31]. Disturbance in cell cycle regulation leads to uncontrolled proliferation of cancer cells. The G1 to S phase transition is a critical moment in the cell cycle. Accelerated G1-S phase transition promotes cell proliferation [32]. Cyclin and CDKs are central molecules in the overall cell cycle regulatory mechanism that drive cell proliferation. Cyclin is a major regulatory protein of the eukaryotic cell cycle process. It regulates the cell cycle by binding to CDKs to form Cyclin-CDKs complex, and it is able to inhibit cell proliferation and thus cause tumor regression [33]. PCNA is a marker of early LUAD [34] and p21 is a suppressor of CDK that indirectly inhibits cell cycle and proliferation [35]. RNH1 reduces the activity and proliferation of LUAD cells by regulating the levels of PCNA, CDK2, Cyclin A and p21. Cell death pathways provide useful clinical insights into cancer [36]. The apoptotic and proliferative phenotypes of LUAD cells are critical for the development of LUAD [37]. LUAD was effectively inhibited when LUAD cell proliferation was suppressed and apoptosis was promoted [38]. Ribonuclease1 treatment resulted in a significant reduction in apoptosis in mouse cardiomyocytes, and RNH1 is a potent ribonuclease inhibitor [39]. The Bcl-2 family includes anti-apoptotic protein (Bcl-xL) and pro-apoptotic protein (Bad). They interact to promote or block Cyt C by regulating the release of mitochondrial membrane permeability, thereby activating or inhibiting the apoptotic pathways [40]. Caspases, which are usually present as inactive enzymes, activate the apoptotic pathway and lead to cell death [41]. RNH1 promotes LUAD apoptosis by regulating the expression of these three proteins.

LUAD is a multi-stage process, with apoptosis, cell proliferation and ablation being early events in cancer progression [42]. Usually, tumor progression requires tumor growth and subsequent development of metastasis [43]. Our results suggest that loss of RNH1 accelerated tumor growth and LUAD metastasis. Apoptosis in LUAD cells leads to inhibition of tumor growth in vitro and in vivo [44]. PCNA is an antigen of proliferating nuclei and is involved in DNA replication and repair, chromatin organization and transcription, and sister chromatid cohesion [45]. Knockdown of RNH1 leads to upregulation of PCNA in tumor tissues, representing an increased proliferative capacity of cells. MMPs are involved in tumor progression, and the expression of MMPs positively correlated with migration and invasion of LUAD cells [46]. E-cadherin, Vimentin, and Twist are epithelial mesenchymal transition (EMT) markers in cancer [47]. EMT is characterized by the absence of epithelial cell markers (E-cadherin) and upregulated expression of mesenchymal cell markers (Vimentin). Changes in the expression of these markers lead to decrease adhesion between transitional cells and adjacent epithelial cells, and increase secretion of enzymes that degrades the extracellular matrix [48]. Twist is known as a master regulator of EMT [49]. RNH1 promotes the expression of E-cadherin and suppresses the expression of Vimentin and Twist. It has been shown that upregulation of long-stranded non-coding RNA promoted invasion and migration of LUAD cells [50]. Downregulation of RNH1 increased cell proliferation, migration and invasion, promoted tumorigenesis and metastasis in bladder cancer in vivo, and altered cell morphology and adhesion by inducing the EMT signaling pathway in bladder cancer cells [8]. RNH1 may play a similar role in LUAD.

We then performed high-throughput mRNA sequencing, and screened out differentially expressed genes. Importantly, KEGG analysis showed that differentially expressed genes were enriched in signaling pathways involved in LUAD. MAPK signaling pathway and PI3K-AKT signaling pathway are reported to promote cell proliferation, migration and invasion of LUAD [51]. p53 signaling pathway is a critical pathway to inhibit the tumor growth of LUAD [52]. It has been confirmed that the dysregulated cell cycle plays a major role in promotion and progression of LUAD [53]. RNH1 overexpression affected these pathways to varying degrees. Our findings suggested that these pathways might be the potential downstream targets of RNH1 in LUAD, but further experiments are needed to verify this speculation.

The findings of this study have to be seen in light of some limitations. First, we demonstrated a negative regulatory mechanism between RNH1 and E2F1 in vitro, while lacking of in vivo data. The in vivo environment is far more complex than in cultured cells, and more in vivo experiments should be conducted to comprehensively investigate regulatory mechanisms between RNH1 and E2F1. Besides, only male mice were selected as subjects was another potential limitation. In the future, we will further explore the gender differences of LUAD between male and female animals.

## Conclusion

Our data suggested that E2F1 upregulation led to the aberrant expression of RNH1, which was transcriptionally repressed by E2F1 binding to the promoter of RNH1. In addition, RNH1 has a tumor suppressor effect in LUAD. Knockdown of RNH1 promoted cell proliferation, tumorigenesis in vivo and EMT process, and drove cancer cell migration and invasion. Overexpression of RNH1 inhibited the development and metastasis of LUAD. These findings enrich the role of RNH1 in LUAD and provide potential avenues for exploring therapeutic strategies for LUAD.

### Electronic supplementary material

Below is the link to the electronic supplementary material.

Supplementary Material 1

Supplementary Material 2

Supplementary Material 3

Supplementary Material 4

## Data Availability

All data generated or analyzed during this study are included in this published article and its supplementary information files.

## References

[1] Reck M, Rabe KF (2017). Precision diagnosis and treatment for Advanced Non-small-cell Lung Cancer. N Engl J Med.

[2] Siegel RL, Miller KD, Fuchs HE, Jemal A (2022). Cancer statistics, 2022. CA Cancer J Clin.

[3] Wang L, Zhang X, Liu Y, Xu S (2020). Long noncoding RNA FBXL19-AS1 induces tumor growth and metastasis by sponging miR-203a-3p in lung adenocarcinoma. J Cell Physiol.

[4] Denisenko TV, Budkevich IN, Zhivotovsky B (2018). Cell death-based treatment of lung adenocarcinoma. Cell Death Dis.

[5] Sarangdhar MA, Allam R. Angiogenin (ANG)-Ribonuclease inhibitor (RNH1) system in protein synthesis and disease. Int J Mol Sci 2021, 22(3). 10.3390/ijms22031287 PMC7866052 33525475

[6] Chen JX, Gao Y, Liu JW, Tian YX, Zhao J, Cui XY (2005). Antitumor effects of human ribonuclease inhibitor gene transfected on B16 melanoma cells. Int J Biochem Cell Biol.

[7] Tang Y, Liu P, Tian Y, Xu Y, Ren F, Cui X, Fan J (2015). Overexpression of ribonuclease inhibitor defines good prognosis and suppresses proliferation and metastasis in human colorectal cancer cells via PI3K/AKT pathway. Clin Transl Oncol.

[8] Xiong D, Liou Y, Shu J, Li D, Zhang L, Chen J (2014). Down-regulating ribonuclease inhibitor enhances metastasis of bladder cancer cells through regulating epithelial-mesenchymal transition and ILK signaling pathway. Exp Mol Pathol.

[9] Yao X, Li D, Xiong DM, Li L, Jiang R, Chen JX (2013). A novel role of ribonuclease inhibitor in regulation of epithelial-to-mesenchymal transition and ILK signaling pathway in bladder cancer cells. Cell Tissue Res.

[10] Li L, Pan XY, Shu J, Jiang R, Zhou YJ, Chen JX (2014). Ribonuclease inhibitor up-regulation inhibits the growth and induces apoptosis in murine melanoma cells through repression of angiogenin and ILK/PI3K/AKT signaling pathway. Biochimie.

[11] Zhu Y, Das K, Wu J, Lee MH, Tan P (2014). RNH1 regulation of reactive oxygen species contributes to histone deacetylase inhibitor resistance in gastric cancer cells. Oncogene.

[12] Gaubatz S, Lindeman GJ, Ishida S, Jakoi L, Nevins JR, Livingston DM, Rempel RE (2000). E2F4 and E2F5 play an essential role in pocket protein-mediated G1 control. Mol Cell.

[13] Lu Z, Luo RZ, Peng H, Huang M, Nishmoto A, Hunt KK, Helin K, Liao WS, Yu Y (2006). E2F-HDAC complexes negatively regulate the tumor suppressor gene ARHI in breast cancer. Oncogene.

[14] Xiang S, Wang Z, Ye Y, Zhang F, Li H, Yang Y, Miao H, Liang H, Zhang Y, Jiang L (2019). E2F1 and E2F7 differentially regulate KPNA2 to promote the development of gallbladder cancer. Oncogene.

[15] Xu TP, Wang YF, Xiong WL, Ma P, Wang WY, Chen WM, Huang MD, Xia R, Wang R, Zhang EB (2017). E2F1 induces TINCR transcriptional activity and accelerates gastric cancer progression via activation of TINCR/STAU1/CDKN2B signaling axis. Cell Death Dis.

[16] Kunigal S, Ponnusamy MP, Momi N, Batra SK, Chellappan SP (2012). Nicotine, IFN-gamma and retinoic acid mediated induction of MUC4 in pancreatic cancer requires E2F1 and STAT-1 transcription factors and utilize different signaling cascades. Mol Cancer.

[17] Pan YC, Li CF, Ko CY, Pan MH, Chen PJ, Tseng JT, Wu WC, Chang WC, Huang AM, Sterneck E (2010). CEBPD reverses RB/E2F1-mediated gene repression and participates in HMDB-induced apoptosis of cancer cells. Clin Cancer Res.

[18] Zhang Q, Shi R, Bai Y, Meng L, Hu J, Zhu H, Liu T, De X, Wang S, Wang J (2021). Meiotic nuclear divisions 1 (MND1) fuels cell cycle progression by activating a KLF6/E2F1 positive feedback loop in lung adenocarcinoma. Cancer Commun (Lond).

[19] Chen R, Xia W, Wang S, Xu Y, Ma Z, Xu W, Zhang E, Wang J, Fang T, Zhang Q (2019). Long noncoding RNA SBF2-AS1 is critical for tumorigenesis of early-stage lung adenocarcinoma. Mol Ther Nucleic Acids.

[20] Lam SK, Li YY, Zheng CY, Leung LL, Ho JC (2014). E2F1 downregulation by arsenic trioxide in lung adenocarcinoma. Int J Oncol.

[21] Gao C, Dong R, Li Y, Liang J, Tian H (2021). MCTS1 promotes the development of lung adenocarcinoma by regulating E2F1 expression. Oncol Lett.

[22] Hu Z, Zhu L, Zhang Y, Chen B (2022). N6-methyladenosine-induced SVIL antisense RNA 1 restrains lung adenocarcinoma cell proliferation by destabilizing E2F1. Bioengineered.

[23] Chen Z, Song Y, Li P, Gao W. GRIN2D knockdown suppresses the progression of lung adenocarcinoma by regulating the E2F signalling pathway. Cell Signal 2023:110685. 10.1016/j.cellsig.2023.110685 37084840

[24] Brambilla E, Gazdar A (2009). Pathogenesis of lung cancer signalling pathways: roadmap for therapies. Eur Respir J.

[25] Spella M, Stathopoulos GT. Immune Resistance in Lung Adenocarcinoma. Cancers 2021, 13(3). 10.3390/cancers13030384 PMC7864325 33494181

[26] Xu F, Huang X, Li Y, Chen Y, Lin L (2021). M(6)A-related lncRNAs are potential biomarkers for predicting prognoses and immune responses in patients with LUAD. Mol Ther Nucleic Acids.

[27] Wang H, Gao J, Zhang R, Li M, Peng Z, Wang H (2020). Molecular and immune characteristics for lung adenocarcinoma patients with CMTM6 overexpression. Int Immunopharmacol.

[28] Luo J, Wang H, Wang L, Wang G, Yao Y, Xie K, Li X, Xu L, Shen Y, Ren B (2021). lncRNA GAS6-AS1 inhibits progression and glucose metabolism reprogramming in LUAD via repressing E2F1-mediated transcription of GLUT1. Mol Ther Nucleic Acids.

[29] Gao Y, Li H, Ma X, Fan Y, Ni D, Zhang Y, Huang Q, Liu K, Li X, Wang L (2017). KLF6 suppresses metastasis of Clear Cell Renal Cell Carcinoma via Transcriptional repression of E2F1. Cancer Res.

[30] Techer H, Koundrioukoff S, Nicolas A, Debatisse M (2017). The impact of replication stress on replication dynamics and DNA damage in vertebrate cells. Nat Rev Genet.

[31] Rucksaken R, Pairojkul C, Pinlaor P, Khuntikeo N, Roytrakul S, Selmi C, Pinlaor S (2014). Plasma autoantibodies against heat shock protein 70, enolase 1 and ribonuclease/angiogenin inhibitor 1 as potential biomarkers for cholangiocarcinoma. PLoS ONE.

[32] Bertoli C, Skotheim JM, de Bruin RA (2013). Control of cell cycle transcription during G1 and S phases. Nat Rev Mol Cell Biol.

[33] Roskoski R (2019). Cyclin-dependent protein serine/threonine kinase inhibitors as anticancer drugs. Pharmacol Res.

[34] Lamort AS, Kaiser JC, Pepe MAA, Lilis I, Ntaliarda G, Somogyi K, Spella M, Behrend SJ, Giotopoulou GA, Kujawa W et al. Prognostic phenotypes of early-stage lung adenocarcinoma. Eur Respir J 2022, 60(1). 10.1183/13993003.01674-2021 34887322

[35] Mansilla SF, de la Vega MB, Calzetta NL, Siri SO, Gottifredi V. CDK-Independent and PCNA-Dependent functions of p21 in DNA replication. Genes (Basel) 2020, 11(6). 10.3390/genes11060593 PMC7349641 32481484

[36] Ahluwalia P, Ahluwalia M, Mondal AK, Sahajpal N, Kota V, Rojiani MV, Rojiani AM, Kolhe R. Immunogenomic Gene Signature of Cell-Death Associated Genes with prognostic implications in Lung Cancer. Cancers 2021, 13(1). 10.3390/cancers13010155 PMC7795632 33466402

[37] Wu Y, Chang N, Zhang Y, Zhang X, Xu L, Che Y, Qiao T, Wu B, Zhou Y, Jiang J (2021). METTL3-mediated m(6)a mRNA modification of FBXW7 suppresses lung adenocarcinoma. J Exp Clin Cancer Res.

[38] Jiang X, Li Y, Zhang N, Gao Y, Han L, Li S, Li J, Liu X, Gong Y, Xie C (2021). RRM2 silencing suppresses malignant phenotype and enhances radiosensitivity via activating cGAS/STING signaling pathway in lung adenocarcinoma. Cell Biosci.

[39] Zechendorf E, O’Riordan CE, Stiehler L, Wischmeyer N, Chiazza F, Collotta D, Denecke B, Ernst S, Muller-Newen G, Coldewey SM et al. Ribonuclease 1 attenuates septic cardiomyopathy and cardiac apoptosis in a murine model of polymicrobial sepsis. JCI Insight 2020, 5(8). 10.1172/jci.insight.131571 PMC7205433 32213712

[40] Yu Y, Zhong Z, Guan Y (2015). The downregulation of Bcl-xL/Bcl-2-associated death promoter indicates worse outcomes in patients with small cell lung carcinoma. Int J Clin Exp Pathol.

[41] Elmore S (2007). Apoptosis: a review of programmed cell death. Toxicol Pathol.

[42] Sainz de Aja J, Dost AFM, Kim CF (2021). Alveolar progenitor cells and the origin of lung cancer. J Intern Med.

[43] Shiwarski DJ, Shao C, Bill A, Kim J, Xiao D, Bertrand CA, Seethala RS, Sano D, Myers JN, Ha P (2014). To grow or go: TMEM16A expression as a switch between tumor growth and metastasis in SCCHN. Clin Cancer Res.

[44] Gao J, Qiu X, Xi G, Liu H, Zhang F, Lv T, Song Y (2018). Downregulation of GSDMD attenuates tumor proliferation via the intrinsic mitochondrial apoptotic pathway and inhibition of EGFR/Akt signaling and predicts a good prognosis in nonsmall cell lung cancer. Oncol Rep.

[45] Cardano M, Tribioli C, Prosperi E (2020). Targeting proliferating Cell Nuclear Antigen (PCNA) as an effective strategy to inhibit Tumor Cell Proliferation. Curr Cancer Drug Targets.

[46] Li Y, Huang H, Ye X, Huang Z, Chen X, Wu F, Lin T (2021). Mir-202-3p negatively regulates MMP-1 to inhibit the proliferation, migration and invasion of lung adenocarcinoma cells. Cell Cycle.

[47] Banyard J, Bielenberg DR (2015). The role of EMT and MET in cancer dissemination. Connect Tissue Res.

[48] De Craene B, Berx G (2013). Regulatory networks defining EMT during cancer initiation and progression. Nat Rev Cancer.

[49] Odero-Marah V, Hawsawi O, Henderson V, Sweeney J (2018). Epithelial-mesenchymal transition (EMT) and prostate Cancer. Adv Exp Med Biol.

[50] Zhang L, Zhou XF, Pan GF, Zhao JP (2014). Enhanced expression of long non-coding RNA ZXF1 promoted the invasion and metastasis in lung adenocarcinoma. Biomed Pharmacother.

[51] Li M, Wu R, Zhu D, Wang L, Liu S, Wang R, Deng C, Zhang S, Chen M, Lu R et al. Nucleophosmin promotes lung adenocarcinoma cell proliferation, migration and invasion by activating the EGFR/MAPK signaling pathway. Oncol Rep 2023, 49(6). 10.3892/or.2023.8563 PMC10196711 37165929

[52] Tang H, Liu J, Huang J (2022). GMFG (glia maturation factor gamma) inhibits lung cancer growth by activating p53 signaling pathway. Bioengineered.

[53] Shan G, Bi G, Bian Y, Valeria B, Zeng D, Zhang H, Yao G, Zhang Y, Fan H, Zhan C (2022). Genomic and Tumor Microenvironment differences between cell cycle progression pathway Altered/Non-Altered patients with lung adenocarcinoma. Front Oncol.

