# Supplementary material for: New link between RNH1 and E2F1: regulates the development of lung adenocarcinoma
Source: BMC Cancer. 2024 May 24;24:635. doi: 10.1186/s12885-024-12392-6 (PMC11118993; doi:10.1186/s12885-024-12392-6)
Supplement: Supplementary file 3 — Supplementary Material 3 [file 12885_2024_12392_MOESM3_ESM.docx]

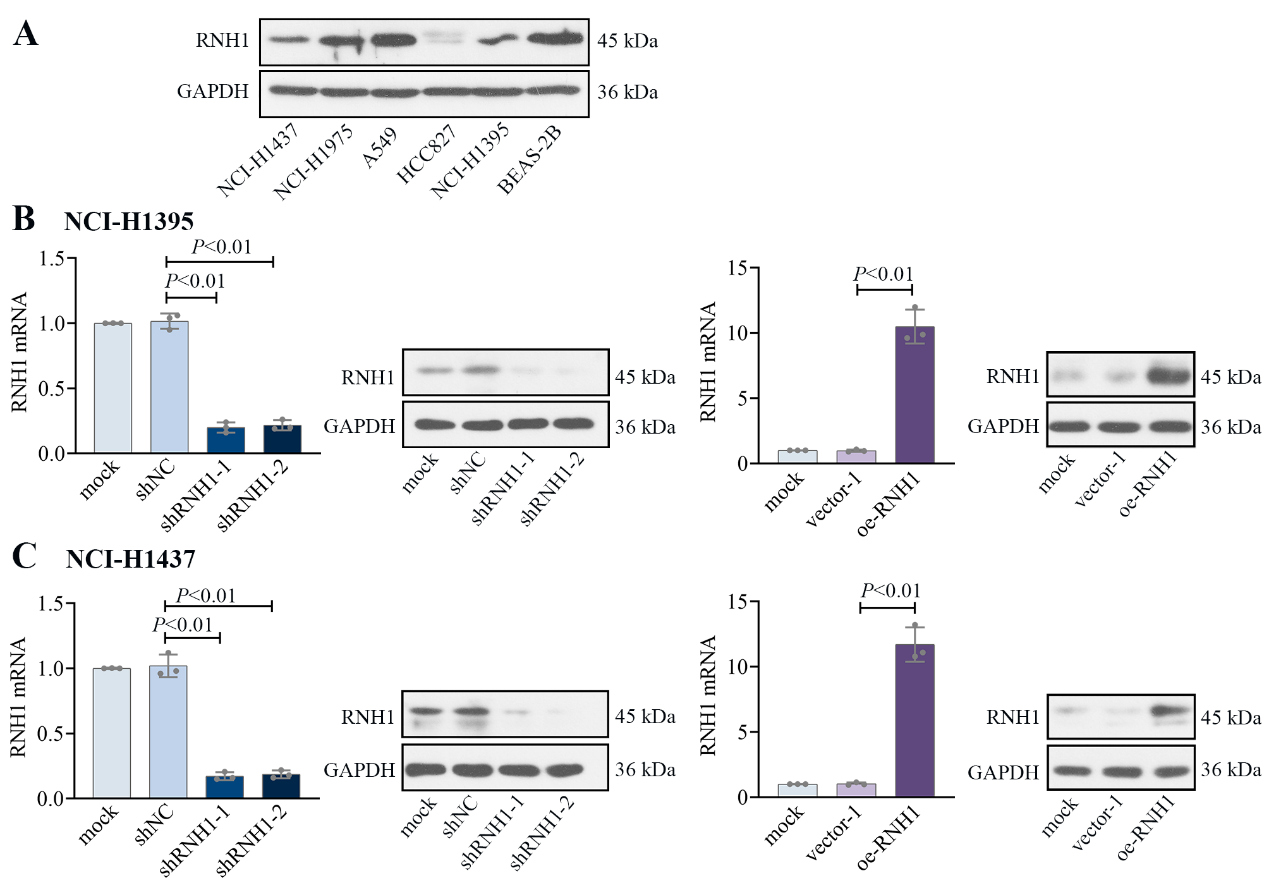


Supplementary Figure S1. RNH1 knockdown and RNH1 overexpression models was successfully constructed in LUAD cells. (A) Western blot was performed to detect the expression of RNH1 in LUAD cell lines (NCI-H1975, NCI-H1395, A549, NCI-H1437, HCC827) and human normal lung epithelial cells (BEAS-2B). qRT-PCR and Western blot were used to detect the expression of RNH1 in NCI-H1395 cells (B) and NCI-H1437 cells (C). shNC, negative control; vector-1, empty vector 1. *P*<0.05 was considered statistically significant.


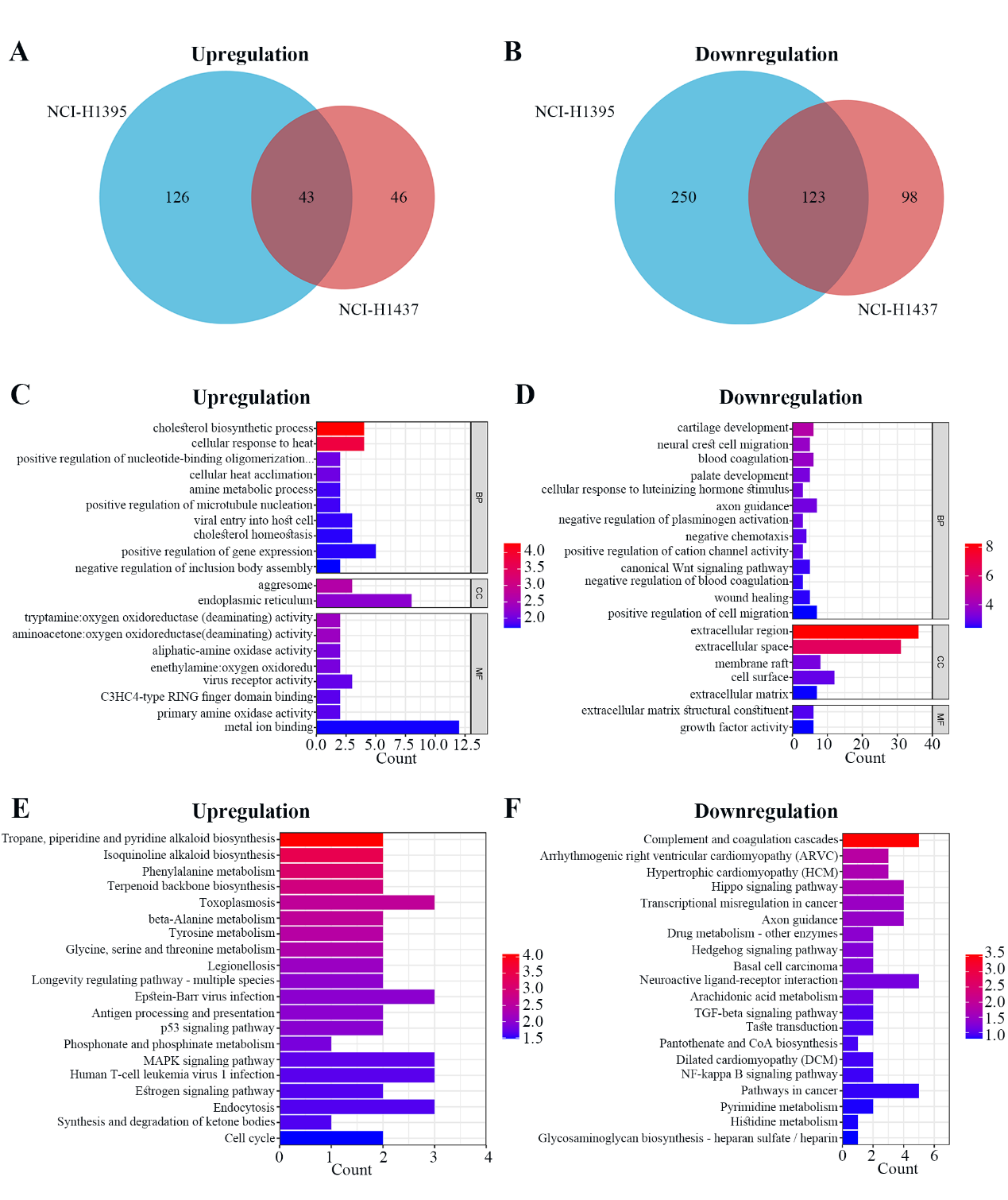


Supplementary Figure S2. Venn plots showing the intersection of up-regulated genes (A) and down-regulated genes (B) in NCI-H1395 cells and NCI-H1437 cells. The number in the Venn diagram indicated the number of genes belonging to the corresponding intersection. GO enrichment analysis was performed on the genes with high expression (C) and low expression (D) selected after intersection. KEGG analysis was performed on the genes with high expression (E) and low expression (F) after intersection selection.


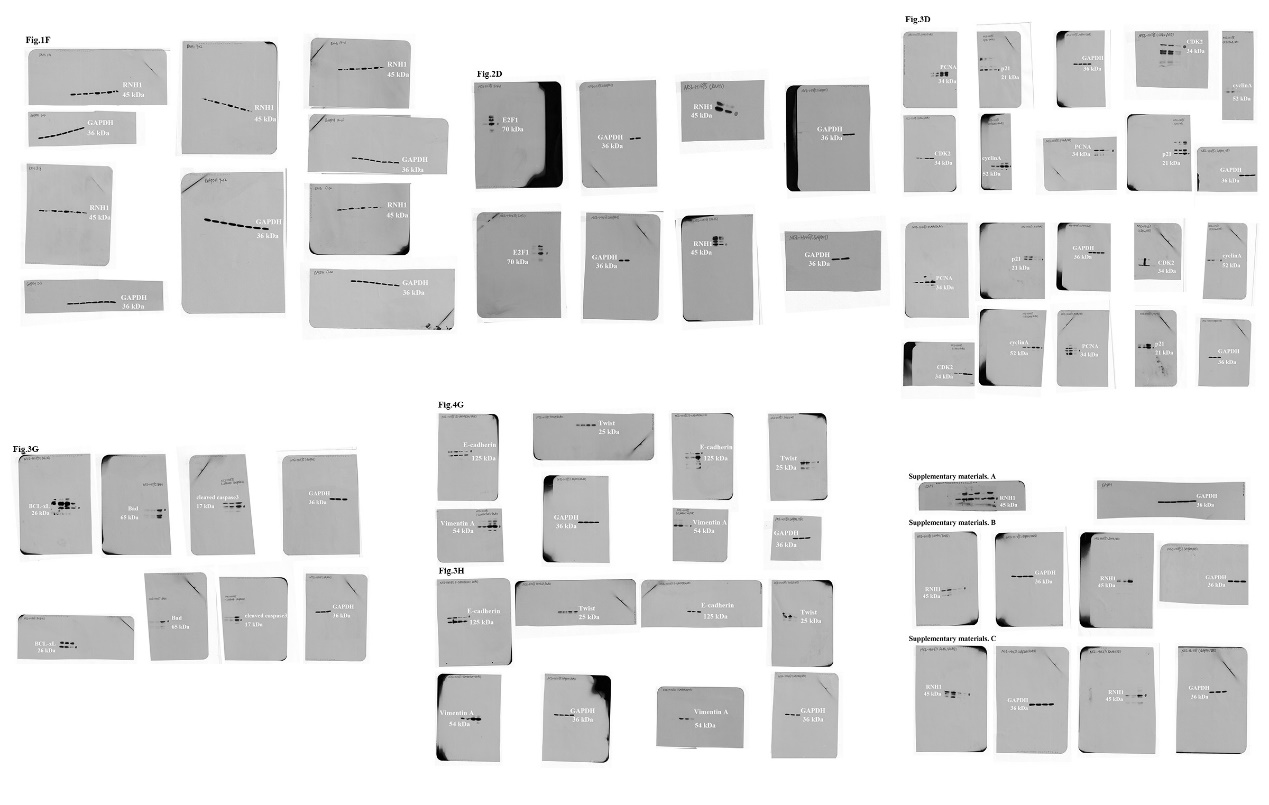


Supplementary Figure S3 Full-length of all blots.

Table.S1 Information about primary antibodies

| Primary antibody | Dilution ratio | | Manufacturer | Number |
| --- | --- | --- | --- | --- |
| RNH1 | | 1: 1000 | Affinity | DF1217 |
| proliferating cell nuclear antigen (PCNA) | | 1: 1000 | Abclonal, Wuhan, China | A12427 |
| cyclin dependent kinase 2 (CDK2) | | 1: 1000 | Abclonal | A0294 |
| Cyclin | | 1: 500 | Abclonal | AF0142 |
| cyclin-dependent kinase inhibitor 1A (p21) | | 1: 500 | Abclonal | A2691 |
| Calcineurin E (E-cadherin) | | 1: 500 | Abclonal | A3044 |
| Vimentin | | 1: 1000 | Affinity | AF7013 |
| Twist | | 1: 1000 | Abclonal | A3237 |
| Bcl-2 associated death promoter (BCL-xL) | | 1: 1000 | Abclonal | A19703 |
| BCL2 associated agonist of cell death (Bad) | | 1: 1000 | Abclonal | A19595 |
| cleaved caspase 3 | | 1: 1000 | Affinity | AF7022 |
| E2F1 | | 1: 1000 | Abclonal | A19579 |
| p-Erk1/2 | | 1：1000 | Abclonal | A22447 |
| Erk1/2 | | 1：500 | Abclonal | A4782 |
| p-MEK1/2 | | 1：500 | Abclonal | AP1349 |
| MEK1/2 | | 1：500 | Abclonal | A4868 |
| p-PI3Kp85/p55 | | 1：1000 | Affinity | AF3242 |
| PI3K | | 1：1000 | Affinity | AF6783 |
| p-Akt | | 1：1000 | Abclonal | AP1208 |
| Akt | | 1：1000 | Abclonal | A17909 |
| MMP9 | | 1:100 | Affinity | AF5228 |
| GAPDH | | 1: 10000 | Proteintech, Wuhan,China | 60004-1-Ig |

Dilution ratio, manufacturer and catalogue number for primary antibodies used in western blot.

Table.S2. Correlation between RNH1 expression and clinicopathological features of LUAD

| Clinicopathological Variables | High (36) | Low (84) | Chi-squared test *P* Value |
| --- | --- | --- | --- |
| Gender |  |  |  |
| Female | 11 | 28 |  |
| Male | 25 | 56 | 0.766 |
| Age, years |  |  |  |
| ＜59 | 15 | 32 |  |
| ≥59 | 21 | 52 | 0.713 |
| Tumor size, cm |  |  |  |
| ≤1.9 | 15 | 43 |  |
| ＞1.9 | 21 | 41 | 0.339 |
| Primary tumor |  |  |  |
| T1 | 25 | 76 |  |
| T2/T3/T4 | 11 | 8 | 0.004 |
| Lymph node metastasis |  |  |  |
| N0 | 30 | 81 |  |
| N1 | 4 | 1 |  |
| N2 | 2 | 2 | **0.028** |
| Histological grading |  |  |  |
| 1 | 10 | 18 |  |
| 2 | 18 | 51 |  |
| 3 | 8 | 15 | 0.552 |
| Clinical stages |  |  |  |
| I | 30 | 81 |  |
| II/III | 6 | 3 | **0.034** |
| TNM stages |  |  |  |
| I | 28 | 79 |  |
| II | 5 | 3 |  |
| III | 3 | 2 | **0.031** |

Bold values meaning statistically significant.
