# Supplementary figures and images for: New link between RNH1 and E2F1: regulates the development of lung adenocarcinoma
Source: BMC Cancer. 2024 May 24;24:635. doi: 10.1186/s12885-024-12392-6 (PMC11118993; doi:10.1186/s12885-024-12392-6)

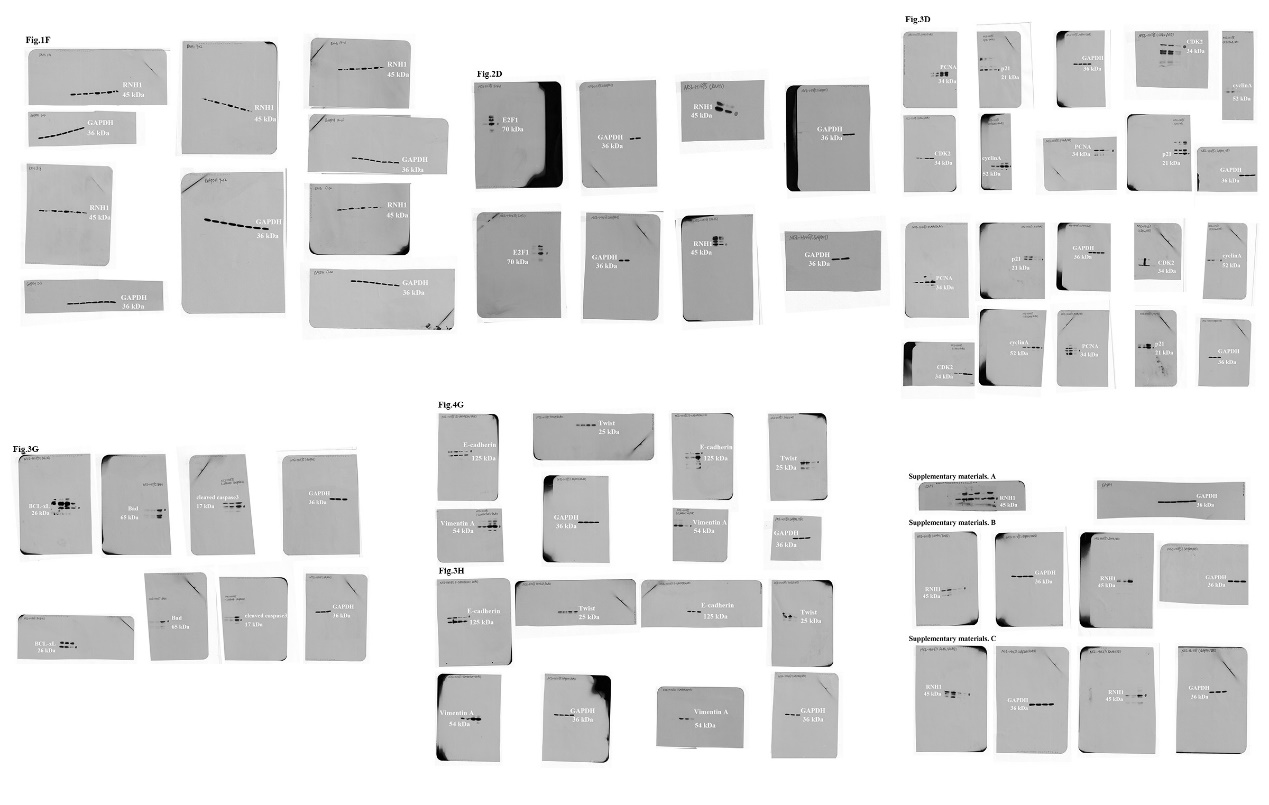


Supplementary Figure S3 Full-length of all blots.

Supplement: Supplementary file 4 — Supplementary Material 4 [file 12885_2024_12392_MOESM4_ESM.docx]
